# Identification of a novel mutation in the *CACNA1C* gene in a Chinese family with autosomal dominant cerebellar ataxia

**DOI:** 10.1186/s12883-019-1381-8

**Published:** 2019-07-10

**Authors:** Jiajun Chen, Yajuan Sun, Xiaoyang Liu, Jia Li

**Affiliations:** Department of Neurology, China–Japan Union Hospital of Jilin University, No 126, Xiantai Street, Changchun, Jilin, 130033 China

**Keywords:** Hereditary ataxia, ADCA, L-type calcium channel, CACNA1C, Mutation

## Abstract

**Background:**

Hereditary ataxia is a group of neurodegenerative diseases with progressive cerebellar ataxia of the gait and limbs as the main symptoms. The genetic patterns of the disease are diverse but it is mainly divided into autosomal dominant cerebellar ataxia (ADCA) and autosomal recessive cerebellar ataxia (ARCA), and about 45 pathogenic loci have been found in ADCA. The purpose of this study was to explore the genetic defect in a Chinese family with ADCA.

**Methods:**

A three-generation Chinese family with ADCA was enrolled in this study, Exome sequencing was conducted in four family members, including the proband, and verified by Sanger sequencing.

**Results:**

The rs779393130 mutation of the *CACNA1C* gene co-segregated with the ataxia phenotype in this family. The mutation was not detected in 50 unaffected controls.

**Conclusions:**

The rs779393130 mutation of *CACNA1C* may be associated with the phenotype of the disease. The *CACNA1C* gene encodes the Cav1.2 (alpha-1) subunit of an L-type calcium channel and this subunit may be related to the ADCA phenotype. These findings may have implications for family clinical monitoring and genetic counseling and may also help in understanding pathogenesis of this disease.

## Background

Hereditary ataxia (HA) is a group of neurodegenerative diseases with progressive cerebellar ataxia of the gait and limbs as the main symptoms; HA affects the cerebellum, brainstem, spinal cord, and cranial nerve nuclei [1]. It is a highly genetically and clinically heterogeneous disease that accounts for about 10 to 15% of nervous system genetic disorders. The genetic patterns of the disease are diverse: mainly autosomal dominant, partly autosomal recessive, and a few cases of X-linked or mitochondrial inheritance [2, 3]. To date, about 45 pathogenic loci have been found in autosomal dominant cerebellar ataxia (ADCA), 35 of which have been cloned, and 70 pathogenic loci have been found in autosomal recessive cerebellar ataxia (ARCA), at least 50 of which have been cloned [4].

Clinical manifestations of the different HA subtypes are similar, making simple diagnosis difficult [5]. Thus, genetic testing has proven to be very valuable for patients with HA. Genetic testing techniques are currently the most efficient tools for HA diagnosis and classification. Genotypic phenotypic correlation analysis can be challenging because of background genetic or environmental factors. Identifying the causative genes can help us understand the pathogenesis of diseases, such as neuronal growth, differentiation, morphogenesis, migration, and tissue formation [6, 7]. The purpose of this study was to explore the genetic defect in a Chinese family with ADCA.

The Cav1.2 protein encoded by the *CACNA1C* gene is the main component of the L-type voltage-gated calcium channel (LTCC). The characteristics of the Cav1.2 channel, such as channel sensitivity, ion selectivity, and drug reaction with calcium ion, are encoded by *CACNA1C*. Polymorphisms in *CACNA1C* are associated with mental disorders, epilepsy, and migraine. It has been reported that the content of Cav1.2 protein in the cerebellum is significantly altered in mice with ataxia and epilepsy. The function of *CACNA1C* gene may be related to ataxia. Consistent with reports that mutations in channel-encoding genes may cause ADCA, we proposed that a novel mutation in *CACNA1C* (rs779393130) may be related to ADCA in this family.

## Methods

### Pedigree and subjects

This study included three generations of a Chinese family with ADCA. All available individuals underwent a thorough neurological examination by two or more experienced neurologists. All patients were diagnosed at the China-Japan Union Hospital of Jilin University. If the individuals exhibited progressive ataxia, they were considered to be affected. Other information on the age of onset, symptoms at the time of onset, magnetic resonance imaging (MRI) results, Montreal Cognitive Assessment (MoCA) scale, and Scale for the Assessment and Rating of Ataxia (SARA), was obtained. Fifty unrelated, ethnically matched controls (male/female: 25/25, age 35.4 ± 8.9 years) without diagnostic features of ADCA were recruited from the same region. The study was conducted in accordance with the Declaration of Helsinki Principles and approved by the ethics committee of the China-Japan Union Hospital of Jilin University. After obtaining informed consent from all subjects, we obtained venous blood samples from affected and unaffected family members (II-1, II-3, II-5, II-6, II-7, III-3, III-4, III-5) and from the 50 unrelated, ethnically matched, unaffected controls for isolation of genomic DNA. The genetic panel for SCA was performed in the proband and his son, and the common mutations and locations of SCA subtypes were excluded by fluorescence labeled capillary electrophoresis fragment analysis. And then, we chose Exome sequencing to explore the genetic defect in this family.

### Whole-exome sequencing and bioinformatics analysis

Whole-exome sequencing was performed in the proband (II-5) and his sibling (II-7), son (III-4), and niece (III-5). A standard phenol-chloroform extraction method was used to extract genomic DNA from peripheral blood. Following the manufacturer’s procedures, more than 1.5 μg of genomic DNA from each sampled individual was cut by using a sonicator (Covaris); enriched, hybridized, and captured on the Agilent SureSelect Human All Exon V5; and sequenced using the Illumina HiSeq 2000 sequencer (Illumina Inc., San Diego, CA, USA). The average sequencing depth was 57.36 x, which provided enough depth to accurately call variants at 97.2% of targeted exome.

Using the Burrows-Wheeler Calibration tool, clean readings without adapters or degraded readings were mapped to the human reference genome (UCSC HG19). Single nucleotide polymorphisms (SNPs) and insertions/deletions were identified by the sequence alignment/map tool, and then the repeated recognition process was read using the Picard tag. We screened all variants against the SNP database, 1000 Genomes project, and the outer NHLBI exome sequencing projects (ESP) 6500. Sorting Intolerant from Tolerant and Polymorphism Phenotyping version 2 were performed for functional prediction. ANNOVAR (Annotate Variation) software was performed to annotate the candidate variants.

Bioinformatics analysis is mainly based on the screening of candidate genes and their relationship with disease phenotypes. Firstly, candidate gene enrichment analysis is carried out. Through GO function enrichment analysis, three results are generated: cell components, biological pathways and molecular functions. Then KEGG pathway enrichment is carried out. Significant enrichment analysis was used to identify the major biochemical metabolic pathways and signal transduction pathways in which mutant genes participated. Then, gene-disease phenotype correlation analysis was performed. Phenolyzer analysis was performed based on the candidate genes and disease/phenotype names obtained from the previous analysis. Candidate genes are mainly based on harmful classification, dominant and recessive pattern screening and other analysis of screened genes, and the disease and genes are input for analysis on the Phenolyzer website. According to the reports in OMIM, NCBI’s ClinVar, GeneReviews and HPRD, the correlation between disease and gene was analyzed. Phenolyzer software scored the correlation of diseases and genes in the database, and ranked the correlation by the final scoring results.

### Direct sanger sequencing

According to the bioinformatics results, we identified the top 3 candidate pathogenic genes: very low density lipoprotein receptor (*VLDLR*), aldehyde dehydrogenase 5 family member A1 (*ALDH5A1*), and calcium voltage-gated channel subunit alpha1 C (*CACNA1C*). Direct Sanger sequencing was then performed using an ABI 3500 sequencer (Applied Biosystems, Foster City, CA, USA) to identify the top three potential causative variants we have set out from bioinformatics analysis in the family. The primer sequences for the pathogenic variants in the genes were designed as follows. For *CACNA1C* (rs779393130): 5′-CCACGGCTTCCTCGAATCTTG-3′ and 5′-GGGAATTTTCCGCTCCGTCTC-3′; for *VLDLR* (NM_003383: exon16:c.2252–8- > AA): 5′-ACCGACTGTCCTTCCCAAAGT-3′ and 5′-CACCAGGAACAACTCTGGCTTA-3′; and for *ALDH5A1* (rs147358733): 5′-TTCTGCAGTTTAAACATTCTAAAAGA-3′ and 5′-TCAGGGTTTCCTATGTTCTCTTC-3′. Mutation Taster (http://www.mutationtaster.org) was applied to predict the function of genetically pathogenic variants. We first performed Sanger sequencing on affected and unaffected family members (II-1, II-3, II-5, II-6, II-7, III-3, III-4, III-5). Then, Sanger sequencing was performed on 50 unaffected individuals to detect the *CACNA1C* gene variant rs779393130.

## Results

### Clinical findings

**Fig. 1:**
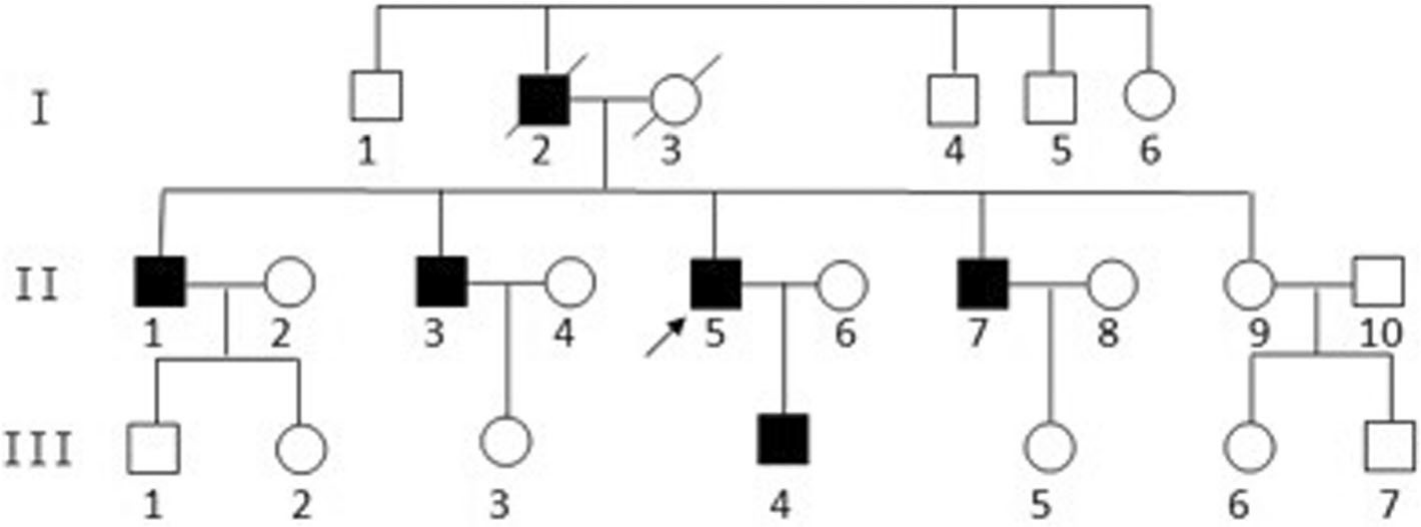
Pedigree of the family

The family we enrolled was a three-generation Chinese family with autosomal dominant inheritance of ataxia; the pedigree is shown in Fig. 1. Exome sequencing was performed in the proband (II-5) and his brother (II-7), son (III-4), and niece (III-5).

The five affected family members alive all developed gait disturbance, and three of them developed difficulties in speech and smooth pursuit defects around the age of 30. They also had disease-causing mutations. The clinical characteristics are shown in Table 1.

**Table 1:** Clinical features of affected family members

Characteristic	II 1	II3	II5	II7	III4
Age at examination	59	53	49	48	26
Age at symptom onset	29	30	28	26	24
Symptoms at onset	gait instability	gait instability	gait instability	gait instability	gait instability
SARA	20	10	12	22	5
Dysarthria	mild	no	mild	moderate	no
Ocular signs	no	no	saccadic pursuit, smooth pursuit defective	saccadic pursuit, smooth pursuit defective	no
Reflexes of			Slight		
Upper limb	normal	normal	hyperreflexia	normal	normal
Lower limb	normal	normal	Slight	normal	normal
			hyperreflexia		
Cerebellar signs in ULs	no	no	moderate	moderate	mild
Gait ataxia	mild	mild	moderate	moderate	mild
Muscle tone	normal	normal	normal	normal	normal
Involuntary movement	no	no	no	no	no
Babinski sign	negative	negative	positive	positive	negative
Cerebral MRI	cerebellar atrophy	NA	cerebellar atrophy	cerebellar atrophy	NA
Other complications	weakness of LLs	no	memory decline	vertigo	no
MoCA	23	24	18	25	28

Abbreviations are as follows: NA, not available; SARA, scale for the assessment and rating of ataxia; UL, upper limb; LL, lower limb. Personal numbers are followed by pedigree numbers according to Fig. 1. Cerebellar signs in ULs means poor distance distinguishing and rotation dysfunction

**Fig. 2:**
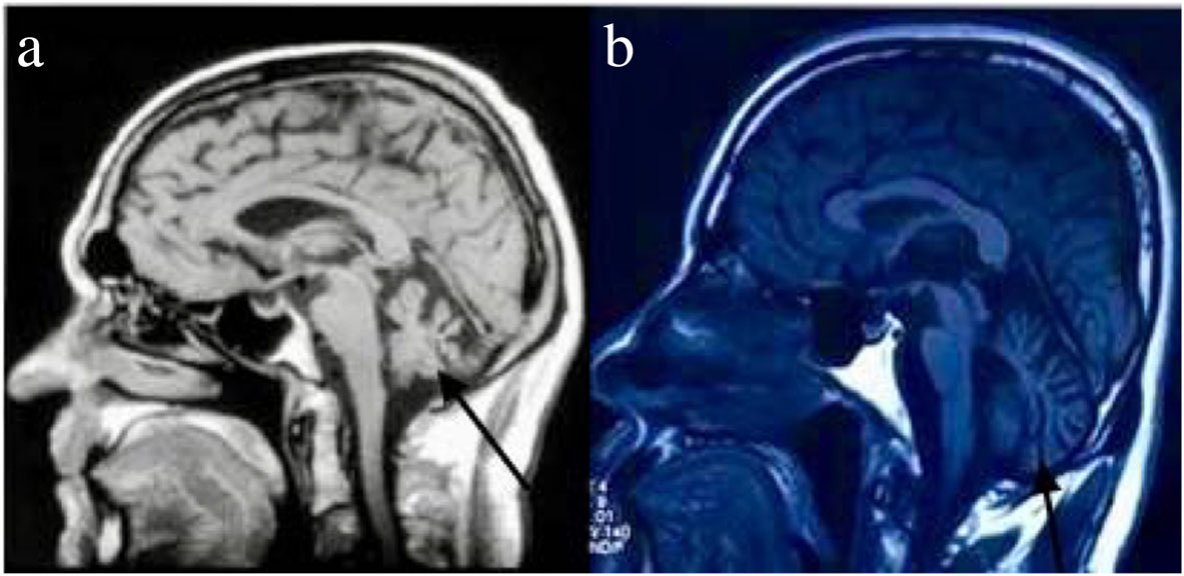
Neuroradiological examination by magnetic resonance imaging (MRI): (**a**) mid-sagittal MRI (T1 weighted image, T1WI) of the brain of patient II-5 (male proband; 49 years old at the time of MRI, 21 years after disease onset; and (**b**) mid-sagittal MRI (T1WI) of the brain of II-7, male sibling of proband; 48 years old at the time of MRI, 22 years after disease onset). The arrows indicate cerebellar atrophy

Neuroradiological examination showed that atrophy was limited to the cerebellum of two individuals we can get (Fig. 2). Therefore, they were diagnosed as pure cerebellar ataxia.

### Exome sequencing

Sequence data quality of four members of this family are shown in Table 2. Known variants with a minor allele frequency (MAF) > 1% identified in dbSNP142, the frequency of 1000 genome plans is > 0.5%, filtering out NHLBI ESP6500. Complex diseases are generally not caused by mutations in a single gene or allele but may involve multiple genes or pathways. Based on, we constructed associations among genes, phenotypes, and diseases by precise algorithms, sequencing results, and multiple databases.

**Table 2:** Data output quality list

Sample name	Raw reads	Raw data(G)	Raw depth (x)	Effective (%)	average read length (bp)
II5	14,438,529	4.33	71.62	99.2	150
II7	14,613,088	4.38	72.45	99.09	150
III4	14,392,960	4.32	71.46	99.23	150
III5	17,271,054	5.18	85.68	98.72	150

**Fig. 3:**
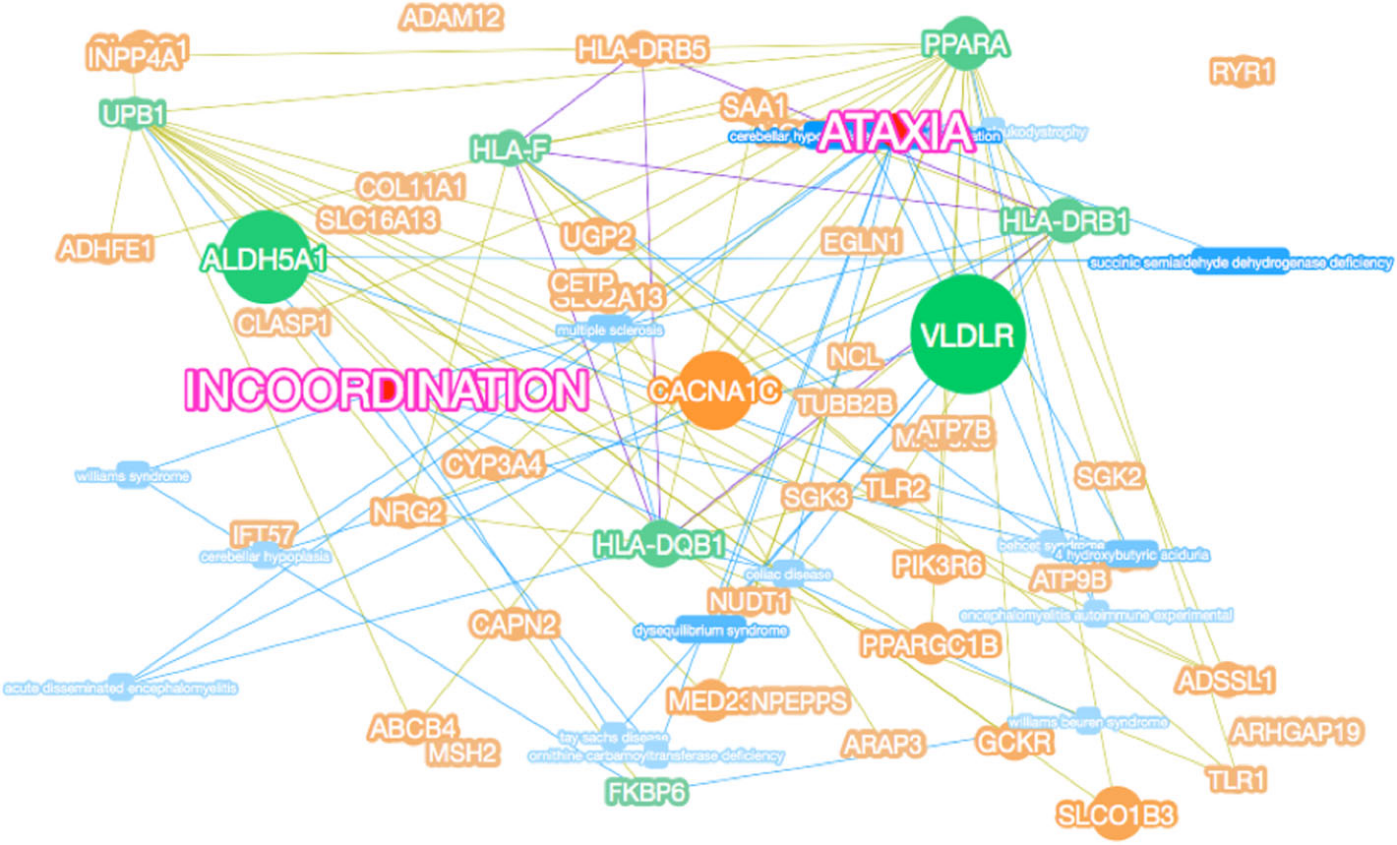
Gene-disease phenotypic association analysis. Words in pink indicate target diseases or phenotypic keywords; blue rounded rectangles indicate related diseases searched by keywords; green dots indicate genes related to diseases in existing reports or databases; orange dots indicate the basis Multiple associations, consider genes associated with green genes

**Fig. 4:**
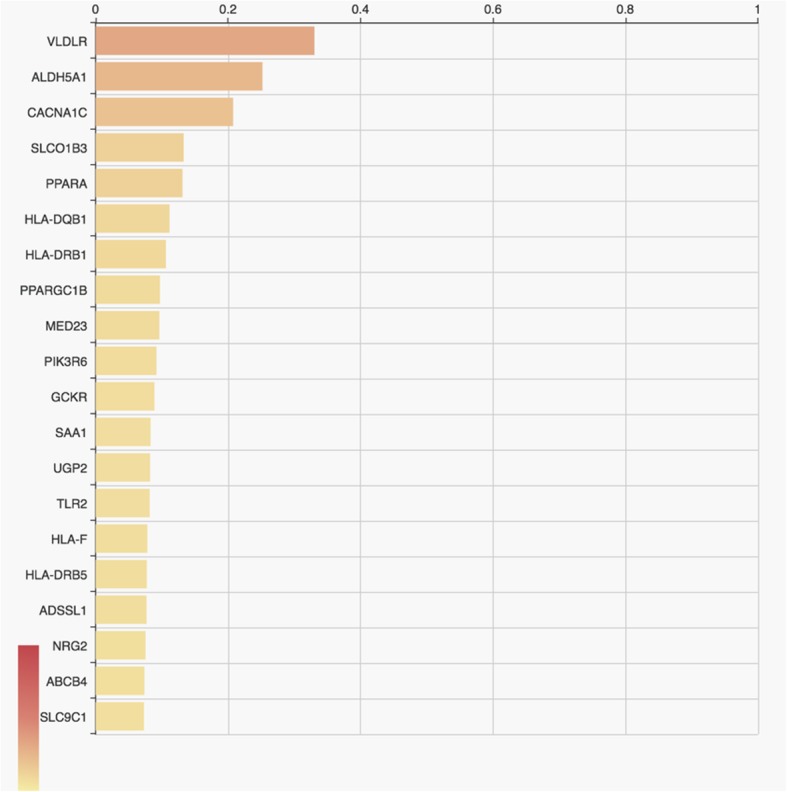
Candidate genes sorted according to their relevance to the disease

Phenolyzer analysis was performed based on candidate genes (genes screened based on harmful classification, dominant and recessive pattern screening, etc.) and disease/phenotype names (diseases and genes were entered into Phenolyzer website for analysis). According to the reports in OMIM, NCBI’s ClinVar, GeneReviews, HPRD and other databases, the correlation between disease and gene was analyzed (Fig. 3). Phenolyzer software scored the correlation based on the information of diseases and genes in the database, and ranked the correlation based on the final scoring results (Fig. 4). From the figures, it can be seen that differences related to ataxia are significant, and genes with strong correlations are *VLDLR*, *ALDH5A1*, and *CACNA1C*, and strong correlations means these correlations between gene variants and ataxia (or phenotype).

### Identification of the pathogenic mutation

As candidate genes selected by the whole-exome sequencing, *VLDLR*, *ALDH5A1*, and *CACNA1C* were sequenced by Sanger sequencing in all affected and unaffected family members. Sanger sequencing showed that only the rs779393130 mutation in *CACNA1C* co-segregated with the disease phenotype in this family (Table 3), and no other differentially expressed genes were found in affected or unaffected family members. Then, Sanger sequencing was performed on 50 healthy individuals to detect the *CACNA1C* variant rs779393130, and no mutation was found at this site in these individuals. The heterozygote *CACNA1C* (rs779393130) variant, which co-segregated with the ataxia phenotype in the family, may be the mutation causing ADCA in this family.

**Table 3:** Genotype analysis

CACNA1C: Single nucleotide polymorphism (SNP) rs779393130			
II-1(patient)	C/G	III-4(patient)	C/G
II-5(patient)	C/G	III-3(normal)	C/C
II-3(patient)	C/G	III-5(normal)	C/C
II-7(patient)	C/G	II-6(normal)	C/C

## Discussion

In this study, we found that a mutation in *CACNA1C*, a gene that encodes the Cav1.2 subunit of an L-type calcium channel, may be related to ADCA in a Chinese family. Dysfunction of ion channels plays a key role in the pathogenesis of ataxia and related diseases. *CACNA1A* (MIM: 601011) was the first channel-coding gene reported to be involved in ADCA. Mutation in *CACNA1A* can lead to spinocerebellar ataxia (SCA) type 6 [SCA6 (MIM: 183086)], episodic ataxia type 2 (MIM: 108500), and familial hemiplegic migraine type 1 (MIM: 141500) [8, 9]. In recent years, *CACNA1G* has been shown to be a pathogenic gene in ADCA [SCA42 (MIM: 604065)] [10–12]. Potassium channel mutations have been described in SCA13 (MIM: 605259) and SCA19/22 (MIM: 607346). Episodic ataxia (EA) is associated with SCA, and mutations in calcium channel genes may lead to EA; for example, mutations in *CACNA1A* and *CACNB4* lead to EA2 (MIM: 108500) and EA5 (MIM: 613855), respectively [13, 14].

The Cav1.2 protein is composed primarily of α1C, α2δ, intracellular β, and CaM subunits, and it plays an important role in the development of dendrites, neuron survival, synaptic plasticity, and memory formation. Studies suggest that Cav1.2 is involved in changes in the calcium permeability of cell membranes, leading to changes in intracellular signaling pathway activity, gene transcription, and synaptic plasticity in the adjustment of advanced brain complex functions such as cognition, emotion, and behavior. Thus, *CACNA1C* is a promising candidate gene for psychiatric disorders, seizures, and migraine [15, 16]. It has been reported, in a mouse model of ataxia and epilepsy, that cerebellar calcium channel proteins such as Cav1.2 were significantly reduced in number, and thus we speculate that CACNA1C is associated with cerebellar ataxia. Based on whole-exome sequencing and Sanger sequencing, we proposed that *CACNA1C* is the causative gene of ADCA in this family [17]. In fact, the rs779393130 SNP is located in an intron, and no pathogenicity reports have been reported. However, our preliminary study found that the SNP co-segregated with the ataxia phenotype in this family. Therefore, we believe that the SNP may be associated with the disease phenotype. Functional validation of animal and cell experiments will be conducted in the future. We will explore the function of the SNP and whether it can affect the phenotype of the disease.

## Conclusions

A combination of clinical evaluation and genetic analyses is recommended in the diagnosis of HA subtypes. Because HA is incurable, it is important to identify the causative genes at additional disease loci to improve patients’ function and quality of life. Further research is needed to elucidate the detailed clinical features of *CACNA1C*-dependent ADCA.

## Data Availability

The data and material used and/or analysed during this study are available from the corresponding author on reasonable request.

## Abbreviations

ADCA: Autosomal dominant cerebellar ataxia
ARCA: Autosomal recessive cerebellar ataxia
EA: Episodic ataxia
HA: Hereditary ataxia
LTCC: L-type voltage-gated calcium channel
